# Continental-Scale Climatic Zones Drive Reorganization of Lake Sediment Microbiome: Diversity, Assembly and Interaction Networks

**DOI:** 10.3390/microorganisms14051013

**Published:** 2026-04-30

**Authors:** Fanjin Ye, Shuai Lu, Yanfang Tian, Pengsong Li, Ziqing Deng, Peng Gao, Hongjie Gao, Xiaoling Liu

**Affiliations:** 1 State Key Laboratory of Environmental Criteria and Risk Assessment, Chinese Research Academy of Environmental Sciences, Beijing 100012, China; yfj0609@163.com (F.Y.); 2108570023005@stu.bucea.edu.cn (S.L.); tian.yanfang@craes.org.cn (Y.T.); 2 Beijing Key Lab for Source Control Technology of Water Pollution, College of Environmental Science and Engineering, Beijing Forestry University, Beijing 100083, China; pengsongli@bjfu.edu.cn; 3 Engineering Research Center for Water Pollution Source Control & Eco-Remediation, College of Environmental Science and Engineering, Beijing Forestry University, Beijing 100083, China; 4 BGI Research, Beijing 100083, China; dengziqing@genomics.cn; 5 BGI, Shenzhen 518083, China; gaopeng@genomics.cn

**Keywords:** climatic gradient, lake sediment, microbial diversity, community assembly, reorganization

## Abstract

Global climate change has altered temperature regimes, hydrological stability, and redox dynamics in inland waters, yet the continental-scale impact of these alterations on sediment microbiomes remains poorly understood. Here, we compiled 562 publicly available 16S rRNA gene datasets from lake sediments across five major climatic zones in China to examine how climatic gradients influence microbial diversity, community assembly, and interaction networks, as well as their associated taxonomic composition and environmental responses. Sediment microbiomes showed clear spatial differentiation in both α- and β-diversity, accompanied by climatic zone-specific taxonomic signatures and biomarker taxa. Community assembly also varied markedly across climatic zones, with stochasticity and dispersal limitation dominating in colder regions, transitional assembly in the south temperate zone, and stronger selective or high-turnover dynamics in the warm subtropics. Importantly, random forest models revealed a clear transition from climate-dominated to anthropogenic-dominated control in sediment microbiome organization: microbial variation in the plateau and temperate regions was primarily associated with climatic and geographic constraints, whereas anthropogenic factors played a more important role in shaping community differentiation in the central subtropical zone. By integrating diversity patterns, taxonomic composition, assembly processes, and network topology, we further propose a three-stage conceptual pattern of sediment microbial community organization along climatic gradients, shifting from a persistence-dominated regime in the cold plateau regions, to an efficiency-dominated regime in the temperate zones, and finally to a plasticity-dominated regime in the warm subtropical regions. These findings would provide a continental-scale framework for understanding sediment microbiome responses to coupled climatic and anthropogenic forcing in inland waters, with implications for future water quality management and ecosystem conservation.

## 1. Introduction

Global climate change has intensified markedly over recent decades, exerting profound impacts on ecosystems through rising temperatures, altered precipitation regimes, and increased hydrological instability [1]. These alterations have accelerated nutrient loading and eutrophication, diminished resilience of inland water ecosystems, and subsequently disrupted their ecological balance and biogeochemical cycles [2,3,4]. As the most climate-sensitive ecosystems, lakes integrate hydrological, climatic, and catchment-scale processes, serving as sentinels of environmental change that reflect both natural variability and anthropogenic disturbances [5]. Therefore, investigating how climatic drivers shape the ecological patterns of lake systems is crucial for assessing the stability and sustainability of freshwater environments under ongoing climate warming. In addition, because lake sediments integrate environmental signals over time, clarifying the climatic controls on modern sediment microbiomes may also help contextualize the ecological meaning of sedimentary records.

Sediments act as both sinks and sources of organic matter, nutrients, and pollutants, thereby mediating the dynamic exchange of materials and energy between the water column and the surrounding landscape [6,7]. Microbial communities inhabiting sediments play central roles in biogeochemical processes, including carbon mineralization, nitrogen transformation, and sulfur cycling, thereby regulating ecosystem functioning and water quality [4,8]. The structure and metabolic activity of these microbial assemblages are governed by a suite of environmental drivers, e.g., temperature, hydrology, oxygen availability, and nutrient status, all of which are directly or indirectly modulated by climatic forcing [8,9]. Thus, as a master variable, climatic variability can reorganize microbial diversity, community composition, and ecological networks, with cascading effects on sediment biogeochemistry and ecosystem resilience.

An increasing body of evidence reveals that climatic conditions exert a dominant influence on microbial communities across diverse ecosystems and spatial scales [10,11,12]. Temperature and precipitation mediate microbial diversity and community composition by modulating organic matter inputs, nutrient availability, and redox conditions [13]. In terrestrial systems, microbial diversity often follows latitudinal or unimodal patterns along climatic gradients, whereas in aquatic ecosystems, climatically driven variations in productivity and oxygen dynamics exert stronger control over microbial community composition [14,15]. Sediment microbiomes, situated at the benthic–pelagic interface of lake ecosystems, integrate chemical and biological signals from overlying waters and catchment inputs, while being influenced by vegetation, land use, and hydrological processes [16]. For instance, increased precipitation enhances the transport of nutrients and organic matter at the terrestrial–aquatic interface, whereas temperature modulates in-lake degradation rates and redox conditions, thereby fostering region-specific microbial communities across climatic zones [17]. Despite a growing body of evidence, most studies remain geographically limited to local or single climatic regions, resulting in a notable scarcity of comprehensive, large-scale syntheses. Hence, continental-scale biogeographic patterns of sediment microbiomes across climatic gradients remain inadequately resolved.

To elucidate the continental-scale climatic effects on sediment microbiomes, we integrated publicly available 16S rRNA gene amplicon data from lake sediments across China to analyze microbial communities within a unified statistical framework. Our specific objectives were to: (1) characterize biogeographic patterns of microbial diversity, community composition, and assembly processes and identify indicator species; (2) assess the relative importance of natural climatic constraints versus anthropogenic drivers using random forest models; and (3) elucidate network topological complexity of microbial interactions. By establishing a mechanistic link between climatic gradients and microbial organization, this study would provide some insights into the resilience mechanisms of inland water ecosystems under global change, while also offering some new knowledge for interpreting microbial variation in lake sediment records.

## 2. Materials and Methods

### 2.1. Basic Information of Five Climatic Zones

**Figure 1:**
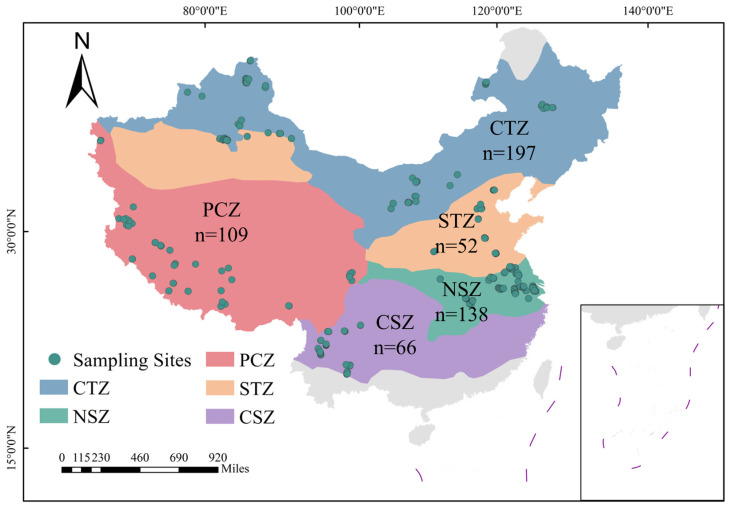
Spatial distribution of five climatic zones and lake sediment sampling sites.

The climatic-zone classification used in this study followed the existing framework issued by the China Meteorological Administration and served as the basis for grouping sampling sites across broad climatic regions in China [18]. Based on this framework, we focused on five predominant climatic zones that encompass most of China’s total lake surface area and number: the plateau climate zone (PCZ), central temperate zone (CTZ), south temperate zone (STZ), northern subtropical zone (NSZ), and central subtropical zone (CSZ) (Figure 1). Together, these zones span a continental-scale climatic gradient from cold, relatively arid plateau settings to warm and humid subtropical environments. Specifically, PCZ is characterized by high elevation and low temperature with generally arid to semi-arid conditions; CTZ represents a cool temperate, strongly continental climate with comparatively limited precipitation; STZ is a temperate transitional zone with milder thermal conditions than CTZ; NSZ is typically warm and humid under monsoonal influence; and CSZ generally exhibits warmer and more humid subtropical conditions than NSZ.

### 2.2. Data Acquisition and Sample Sites

**Figure 2:**
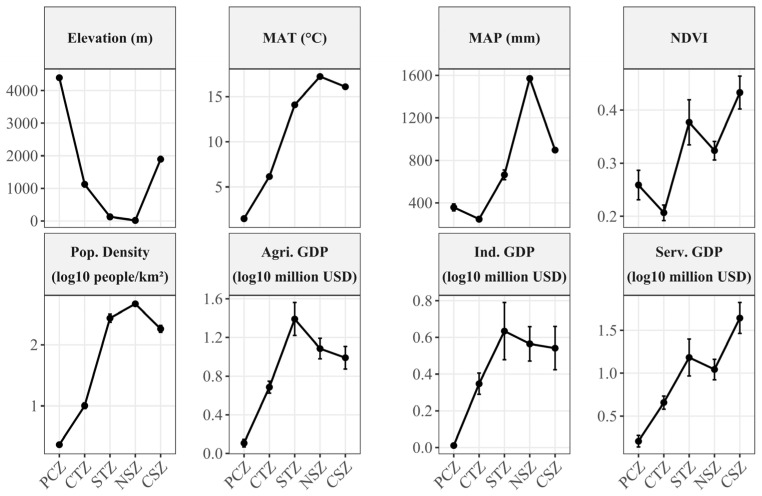
Variation of key environmental and socioeconomic factors across climatic zones. Error bars represent 95% confidence intervals.

A systematic retrieval of 16S rRNA gene amplicon datasets from lake sediments across five continental-scale climatic zones in China was conducted through the Sequence Read Archive (SRA) of the National Center for Biotechnology Information (NCBI) using the search query: “(China OR Chinese) AND (lake) AND (sediment OR deposit) AND (16S rRNA)”. Initially, 4700 publicly available 16S rRNA gene datasets were screened. To reduce potential spatial and methodological heterogeneity associated with publicly available datasets, stringent inclusion and exclusion criteria were applied as follows: (1) datasets generated on Illumina paired-end platforms to ensure high sequencing depth and consistency; (2) the hypervariable V4 region of the 16S rRNA gene (including V4, V3–V4, and V4–V5) for optimal taxonomic resolution and minimizing amplification biases [19,20]; (3) samples with documented GPS coordinates to enable precise integration with climatic metadata; (4) surface sediment samples collected from June to October to reduce variability due to stratification and seasonality; (5) surface sediment samples collected between 2016 and 2022 to constrain the temporal window of observation and improve comparability among datasets; and (6) exclusion of aquaculture-associated water bodies (e.g., fish ponds) to avoid confounding effects from anthropogenic inputs. Following these criteria, 562 16S rRNA gene datasets from lake sediment samples were retained for analysis in our study (Figure 1; Table S1). Environmental and anthropogenic variables of each sampling site were extracted in ArcGIS (v10.8) based on recorded geographic coordinates (Figure 2). Meteorological data, including mean annual temperature (MAT) and mean annual precipitation (MAP), were retrieved from the Environmental Meteorological Data Service Platform (http://eia-data.com/, accessed on 30 August 2025); Elevation was derived from the digital elevation model (DEM), population density, and the normalized difference vegetation index (NDVI) were obtained from the RESDP [18]. Furthermore, socioeconomic intensity was characterized by extracting gross domestic product (GDP) values for industrial, service, and agricultural sectors from the SectGDP30 v2.0 global map at 30″ resolution [21]. As shown in Figure 2, these climatic zones delineate pronounced and systematic environmental gradients. A consistent upward trend in both MAT and MAP is observed from the CTZ toward the humid NSZ. Concurrently, these zones exhibit a marked increase in population density, coupled with a corresponding decline in elevation. These variables were used as broad-scale climatic, geographic, and anthropogenic descriptors of the sampling sites for comparative analysis.

### 2.3. Sequence Processing and Taxonomic Annotation

Raw sequence data from NCBI were treated using the SRA-Toolkit tool (version 3.0.2) based on the corresponding SRR accession numbers. Quality control and adapter trimming were carried out by the fastp tool (version 0.20.0) with a sliding-window approach (Phred ≥ 20) [22]. Primer sequences were identified and removed with the cutadapt tool [23]. Paired-end reads were merged when forward and reverse reads overlapped by at least 20 bp, and unassembled reads were discarded. Denoising and amplicon sequence variant (ASV) inference were then performed using the plugin of divisive amplicon denoising algorithm 2 in the Quantitative Insights Into Microbial Ecology 2 (version 2025.4), which also performed consensus-based chimera removal [24]. Taxonomy was assigned using a naïve Bayes classifier trained on the SILVA reference database (release 138), and non-bacterial sequences were removed from the dataset [25]. Although ASVs were retained as the basic sequence units, downstream ecological analyses were conducted at the genus level to mitigate potential biases arising from primer-specific amplification and variable amplicon lengths across studies, thereby enhancing cross-study comparability. Because taxonomic resolution from short-read 16S rRNA amplicon data is often more robust at the genus level than at finer ranks, genus-level aggregation was used here as a compromise between taxonomic detail and analytical consistency. Subsequently, to account for uneven sequencing depths across datasets, all samples were rarefied to 16,000 reads per sample prior to diversity analyses.

### 2.4. Statistical Analyses

All statistical analyses were conducted using the R computing environment (version 4.5.2). Alpha diversity indices (Shannon, Chao1, and Pielou’s evenness) were calculated using the vegan package, and phylogenetic diversity (PD) was computed using the picante package. Differences in alpha diversity and environmental variables across climatic zones were assessed using the Kruskal–Wallis H test, with Dunn’s post-hoc test and Bonferroni correction for multiple comparisons. Beta diversity was assessed based on Bray–Curtis dissimilarity and weighted/unweighted unifrac distances. Principal Coordinate Analysis (PCoA) was performed to visualize community separation. The significance of community dissimilarity across climatic zones was tested using Permutational Multivariate Analysis of Variance (PERMANOVA) with the adonis2 function in the vegan package (999 permutations). To identify specific bacterial taxa driving the differences among different climatic zones, Linear Discriminant Analysis Effect Size (LEfSe) was performed using the microeco package, with a logarithmic LDA score threshold of 4.0 and an alpha value of 0.05 for the Kruskal–Wallis test [26]. Community assembly mechanism was disentangled using a dual-model approach: (1) the Neutral Community Model (NCM) was used to estimate the contribution of stochastic processes based on the frequency–abundance relationship [27]; (2) the infer Community Assembly Mechanisms by Phylogenetic-bin-based null model analysis (iCAMP) framework was employed to quantify the relative importance of homogeneous selection, heterogeneous selection, homogenizing dispersal, dispersal limitation and undominated processes or drift [28].

To rigorously quantify the non-linear contributions of climatic and anthropogenic drivers to microbial diversity and community assembly, Random Forest (RF) regression models were performed using the rfPermute package. Models were trained with 1000 trees (ntree = 1000) to ensure stabilization of the out-of-bag error estimate. The statistical significance of predictor importance, measured as % increase in mean squared error (%IncMSE), was evaluated through 999 permutations under the null hypothesis of no association between the predictors and the response variable. Furthermore, Partial Dependence Plots (PDPs) were generated using the pdp package to illustrate the marginal relationship between the most significant predictors and the response variables. These analyses were intended to evaluate broad ecological associations between sediment microbiome patterns and site-level climatic, geographic, and anthropogenic factors. Microbial co-occurrence networks were inferred for each climatic zone using the Weighted Gene Co-expression Network Analysis (WGCNA) package. To ensure robust and statistically significant associations, only pairwise correlations with a Spearman’s coefficient ∣*ρ*∣ ≥ 0.6 and a *p* ≤ 0.001 were retained. Network visualization was conducted using Gephi (version 0.9.7). To identify potential keystone taxa, the topological roles of individual nodes were classified according to their within-module connectivity (Zi) and among-module connectivity (Pi). Nodes were categorized into four ecological roles: module hubs (Zi ≥ 2.5, Pi < 0.62), connectors (Zi < 2.5, Pi ≥ 0.62), network hubs (Zi ≥ 2.5, Pi ≥ 0.62), and peripherals (Zi < 2.5, Pi < 0.62) following the criteria established by Olesen et al. (2007) [29]. All statistical plots were generated using the ggplot2 package in R (version 4.5.1), and spatial maps were produced using ArcGIS (version 10.8).

## 3. Results and Discussion

### 3.1. Variation of Sediment Microbial Diversity with Continental-Scale Climatic Gradient

**Figure 3:**
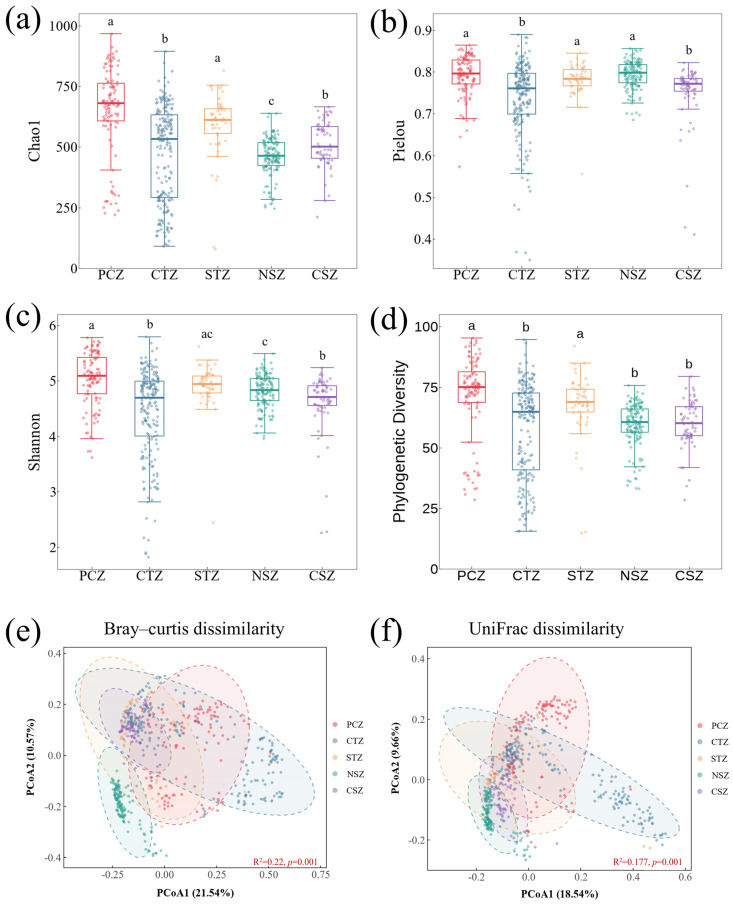
Variations in sediment microbial α- and β-diversity across climatic zones. (**a**–**d**) genus-level alpha diversity indices, and (**e**,**f**) PCoA ordinations based on Bray–Curtis and unifrac dissimilarities across climatic zones. Different letters in boxplots denote significant differences at *p* < 0.05.

Sediment microbial α-diversity at the genus level exhibited significant climatic differences among these five study regions (Figure 3a–d). These climatic zones encompass pronounced gradients in elevation, MAT, and MAP, accompanied by systematic shifts in vegetation cover (quantified as NDVI) and human activity indices (Figure 2). Chao1 richness was highest in the PCZ and remained relatively high in the STZ, whereas the NSZ showed the lowest richness (Figure 3a). Phylogenetic diversity was also elevated in the PCZ and STZ, while both the NSZ and CSZ were characterized by comparatively low and statistically similar PD values (Figure 3d). In contrast, Shannon diversity and Pielou evenness index showed relatively limited variation across the climatic gradient (Figure 3b,c), suggesting that the variation in taxonomic richness, rather than evenness, was the primary contributor to overall α-diversity variation [30]. The elevated taxonomic richness and phylogenetic breadth observed in the PCZ—coinciding with the lowest MAT and highest elevation (Figure 2)—might reflect a low-temperature preservation mechanism, whereby reduced metabolic rates and weakened competitive exclusion may allow more stress-tolerant or dormant lineages to persist [31,32]. Notably, the STZ also exhibited high richness and PD despite intermediate MAT. This pattern is consistent with its role as a temperate–subtropical transition zone where increased precipitation and enhanced vegetation cover might expand niche availability and promote habitat heterogeneity [33].

Climatic zone further structured sediment microbial communities, as revealed by PCoA analysis based on Bray–Curtis and unifrac distances (Figure 3e,f). PCoA1 accounted for 21.54% and 18.54% of the total variation in Bray–Curtis and unifrac dissimilarities, respectively. PERMANOVA further demonstrated that climatic zone alone explained 22.0% and 17.7% of the overall beta-diversity variation using Bray–Curtis and unifrac distance, respectively (*p* = 0.001). The CTZ exhibited the greatest dispersion in ordination space, consistent with its extensive geographic coverage and environmental heterogeneity, whereas microbial communities in the NSZ formed a more compact cluster, suggesting stronger community homogenization under a relatively narrow warm–humid climatic regime (Figure 2). Collectively, the concordant taxonomic and phylogenetic patterns suggest that the temperature–precipitation gradient is one of the critical factors shaping the continental-scale biogeographic organization of surface-sediment microbiomes.

### 3.2. Patterns of Taxonomic Architecture and Region-Specific Keystone Taxa Along Climatic Gradients

**Figure 4:**
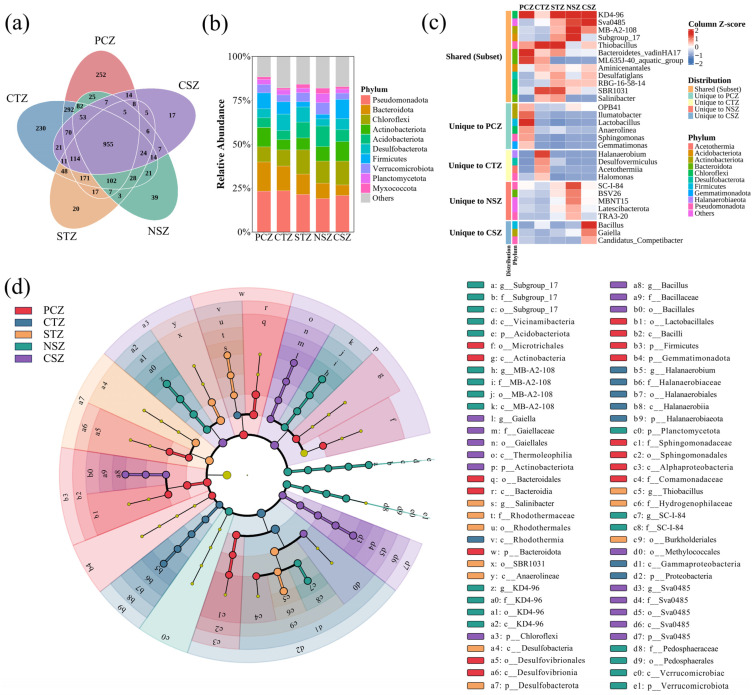
Taxonomic composition, shared and unique genera, and biomarker taxa of sediment microbiomes. (**a**) Shared and unique genera across climatic zones; (**b**) relative abundance of dominant bacterial phyla; (**c**) Z-score standardized heatmap of the top 10 genera across climatic zones; (**d**) cladogram of taxa significantly enriched in different climatic zones identified by LEfSe (LDA score > 4.0, *p* < 0.05).

At the continental scale, sediment microbiomes differed clearly in taxonomic composition among climatic zones (Figure 4). Venn analysis at the genus level revealed a large shared core microbiome, with 955 genera detected across all five climatic zones (Figure 4a). Nevertheless, each zone also contained unique genera, with the highest numbers observed in the PCZ and CTZ, followed by the NSZ, STZ, and CSZ. These results indicate that lake sediment microbiomes across China are structured by a broadly distributed taxonomic core together with region-specific components, suggesting that climatic gradients reorganize communities mainly through differential enrichment and regional filtering. These differences were further reflected in both the relative abundance of dominant phyla and the identity of zone-enriched biomarker taxa. At the phylum level, the sedimentary microbiome was predominantly structured by a conserved core assemblage of Pseudomonadota, Bacteroidota, and Chloroflexi (Figure 4b). However, the relative abundance of these dominant phyla exhibited systematic shifts along the latitudinal gradient. This pattern may indicate differences among climatic zones in the dominant modes of organic matter degradation and redox-related energy metabolism [34]. In the PCZ, microbes were characterized by a distinct dominance of Bacteroidota and Pseudomonadota taxa. These taxa have frequently been associated with the degradation of complex organic matter [35,36]. In contrast, a pronounced increase in the relative abundance of Chloroflexi was observed in warmer temperate to subtropical regions. This increase might be related to environmental conditions more commonly observed in warmer lakes, including stronger stratification and a higher likelihood of low-oxygen sediment microenvironments [37]. These conditions may be associated with anaerobic carbon remineralization and sulfur-related energy metabolism [38]. Notably, Firmicutes exhibited a bimodal distribution across the five major climatic zones, with elevated abundances in both the cold PCZ and the warm CSZ. This pattern suggested a functional convergence under contrasting climatic extremes, wherein spore-forming capability may be selectively advantageous: conferring resilience to prolonged cold stress in the northern regions, while enabling rapid colonization of ephemeral niches in warmer southern zones [39].

A finer-resolution analysis of the top 10 genera (Figure 4c), supported by LEfSe biomarker identification (Figure 4d), further elucidated these potential ecological associations. Even among genera shared by multiple climatic zones, their relative abundances varied across regions. KD4-96 was detected across several climatic zones, but its relative abundance was much lower in the CTZ, whereas LEfSe identified it as a biomarker of the NSZ. This pattern may be related to the distinct hydrochemical conditions of many CTZ lakes, especially their relatively high salinity and alkalinity [40]. Conversely, *Thiobacillus*, a genus comprising known sulfur-oxidizers, was significantly enriched in both the CTZ and STZ. This enrichment was also consistent with the high LDA scores of Hydrogenophilaceae and may indicate that redox interfaces in seasonally stratified lakes, which are common in many temperate regions, favor sulfur-oxidizing taxa [41]. Notably, no single genus among the top 10 was uniquely dominant in the STZ. This pattern is consistent with the transitional character of this zone, where the most abundant taxa were largely shared with adjacent climatic zones rather than forming a clearly distinct set of dominant genera.

The environmental conditions of the PCZ appeared to selectively favor microbial communities characterized by stress-tolerance traits. Sediments within the PCZ were characterized by a specific guild including *Ilumatobacter*, *Gemmatimonas*, and *Sphingomonas*. These genera, identified as biomarkers associated with Actinobacteria and Sphingomonadales, have been frequently reported in the published literature as possessing robust DNA repair mechanisms and metabolic versatility adapted to high-ultraviolet radiation and oligotrophic environments [42]. The co-enrichment of lactic acid bacteria, including fermentative lineages related to Lactobacillales, may suggest a reliance on anaerobic fermentative pathways that help sustain basal microbial activity when prolonged ice cover limits respiratory electron acceptors [39,43].

In the CTZ, the microbial community composition reflected the combined effects of elevated evaporation rates and distinctive mineralogical characteristics. The community was enriched in taxa commonly associated with elevated salinity and redox heterogeneity, notably *Halanaerobium*, *Halomonas*, and *Desulfovermiculus*. This assembly was supported by LEfSe analysis results, which revealed elevated discriminant scores for Halanaerobiaeota and Gammaproteobacteria as shown in Figure 4c. This explained that the sedimentary environment was possibly influenced by sulfur-enriched and potentially hypersaline conditions. In these semi-arid temperate basins, the observed microbiome structure may suggest a stronger association with sulfur- and carbon-related microbial functions, wherein halotolerant fermentative and sulfate-reducing microorganisms may be more prominent than in the freshwater systems of humid regions [44].

Approaching the subtropical maxima in the NSZ and CSZ, the microbiome underwent a structured reorganization favoring anaerobic hydrolysis and enhanced metabolic plasticity, likely in response to higher productivity. In the NSZ, the microbial community was characterized by the enrichment of Acidobacteriota (specifically Subgroup_17) and Verrucomicrobiota, along with BSV26, all serving as biomarkers. This enrichment aligns with the region’s high allochthonous plant inputs, as previous studies have associated these taxa with the degradation of complex polysaccharides under micro-oxic sedimentary conditions [45]. In contrast, the microbial community in the CSZ exhibited a distinct signature dominated by *Bacillus* and Sva0485 (Desulfobacterota), both identified as significant biomarkers. The increased relative abundance of *Bacillus* in these warmest climatic zones may reflect an adaptive strategy to accelerated organic turnover. Members of this genus are often associated with the production of extracellular hydrolytic enzymes, suggesting a potential role in the processing of complex organic substrates in warm and productive lake sediments [46]. This metabolic versatility may be advantageous in warmer, productive lakes, where intense microbial respiration can generate transient anoxic niches and favor taxa with flexible metabolic strategies.

Collectively, these biogeographic patterns suggest that climatic zone functioned as a broad environmental filter, favoring traits associated with persistence under energy-limited conditions on plateaus, specialized redox metabolisms in the heterogeneous temperate zones, and metabolic plasticity in the energy-rich subtropical regions. This gradient-driven sorting mechanism underscored the pivotal role of climate in structuring the taxonomic composition of lake sediment communities and suggested potential differences in the ecological strategies associated with microbial functioning across climatic zones.

### 3.3. Community Assembly Mechanisms of Sediment Microbiomes Across Climatic Gradients

**Figure 5:**
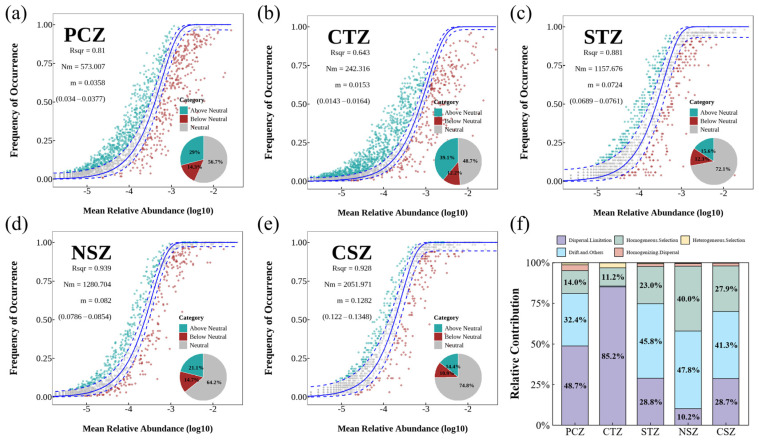
Neutral model fitting and ecological assembly processes across climatic zones. (**a**–**e**) Sloan’s neutral models with 95% confidence intervals; (**f**) relative contributions of deterministic and stochastic processes inferred from iCAMP. The blue solid line represents the predicted occurrence frequency under the neutral model, and the blue dashed lines indicate the 95% confidence intervals.

To decipher the ecological mechanism underlying the assembly of sediment microbial communities, a dual-framework analytical approach that integrated the NCM with the iCAMP-based process-partitioning analysis was used (Figure 5). These models collectively indicated that the balance between stochastic (e.g., dispersal and ecological drift) and deterministic (e.g., environmental selection) assembly processes varied markedly across climatic zones [27,28].

In the PCZ, community assembly was primarily governed by stochastic processes. The NCM achieved a moderate goodness-of-fit (R^2^ = 0.81), while iCAMP partitioning revealed that dispersal limitation (48.7%) and stochastic drift (32.4%) collectively explained over 80% of the observed community turnover (Figure 5f). This prevalence of stochasticity in this region was likely attributable to the distinctive environmental constraints inherent to the plateau: persistent low temperatures and oligotrophic conditions, which can suppress microbial metabolic activity and reduce population sizes, thereby theoretically enhancing the influence of ecological drift [47,48]. Moreover, the complex topography of the plateau might impose physical barriers limiting microbial migration, facilitating community divergence through passive accumulation of stochastic differences rather than deterministic environmental filtering [49,50].

In the CTZ, microbial community assembly was characterized by a predominance of regional isolation. This zone exhibited the lowest fit to the neutral model (R^2^ = 0.64) and the lowest estimated migration rate (Nm = 242), with iCAMP analysis revealing a dominant role of dispersal limitation (85.2%). These results suggest that the microbial community turnover at the regional scale was primarily constrained by physical boundaries. This pattern aligned with the landscape configuration of the temperate zone, where lakes often existed as discontinuous habitats embedded within semi-arid or topographically complex matrices [51]. This pronounced dispersal limitation implied that regional-scale physical barriers likely limit species dispersal and gene flow across the CTZ. Although environmental filtering might still operate at the local scale within individual lakes, the limited connectivity appeared to be the primary driver of community turnover at the regional scale [52].

In the STZ, the microbial community assembly displayed a transitional signature. In this region, the constraints imposed by geographic isolation were attenuated, as evidenced by a markedly improved fit to the neutral model (R^2^ = 0.88) and an elevated migration rate (Nm = 1157). iCAMP-based partitioning revealed a mixed assembly mechanism, predominantly governed by stochastic drift (45.8%), but counterbalanced by substantial contributions from homogeneous selection (23.0%) and dispersal limitation (28.8%). This hybrid assembly pattern suggested that increasing precipitation and potentially attenuated physical barriers, relative to the CTZ, facilitated enhanced microbial dispersal and connectivity. Nevertheless, persistent moderate selective pressures maintained a degree of deterministic control, precluding a purely stochastic assembly regime [53].

As climatic energy input increased toward the subtropics, microbial community assembly mechanisms reorganized into a dynamic interplay between deterministic filtering and high-turnover neutrality. Within this gradient, the NSZ exhibited the strongest deterministic signal, with homogeneous selection contributing 40.0% to community assembly, the highest proportion observed across all zones. This surge in selection pressure corresponded to the environmentally dynamic conditions inferred for this region (Section 3.2), suggesting that relatively consistent resource inputs and micro-oxic habitats may impose a strong environmental filter, thereby favoring specific microbial guilds [53,54].

Finally, within the CSZ, the system transitioned into a regime characterized by high dispersal rate and stochastic drift. The NCM showed a high goodness-of-fit in the CSZ (R^2^ = 0.93) and the highest migration potential (Nm = 2052), suggesting highly efficient dispersal in this zone. iCAMP-based partitioning analysis revealed a mixed dominance of stochastic drift (41.3%) and dispersal limitation (28.7%), accompanied by a moderate contribution from selection (27.9%). The prevalence of metabolically versatile taxa observed in this zone (Section 3.2) aligned with this pattern, where warm, eutrophic, and interconnected waters facilitate a mass effect—characterized by high growth rates and frequent propagule exchange—that promotes regional homogenization and counteracts local selective gradients [55,56].

In summary, microbial community assembly in lake sediments exhibited a three-stage transition across the climatic zones examined here: (1) dominance of stochastic processes and dispersal limitation in the colder regions (PCZ, CTZ); (2) a transitional balance between stochastic and deterministic processes in the STZ; and (3) divergent assembly features in the warmer zones, with the strongest contribution of homogeneous selection in the NSZ and high dispersal potential coupled with drift-dominated neutral dynamics in the CSZ. Under a space-for-time perspective, this observed gradient may suggest a potential trajectory in which future climate warming could drive lake sediment microbiomes from dispersal-limited, structurally isolated assemblages towards more interconnected community regimes. However, such projections must be interpreted with caution, as they are contingent upon the assumption that future climate warming will parallel existing spatial climatic gradients and will not be accompanied by novel anthropogenic stressors capable of disrupting these natural assembly patterns.

### 3.4. Climatic and Anthropogenic Drivers of Microbial Differentiation

**Figure 6:**
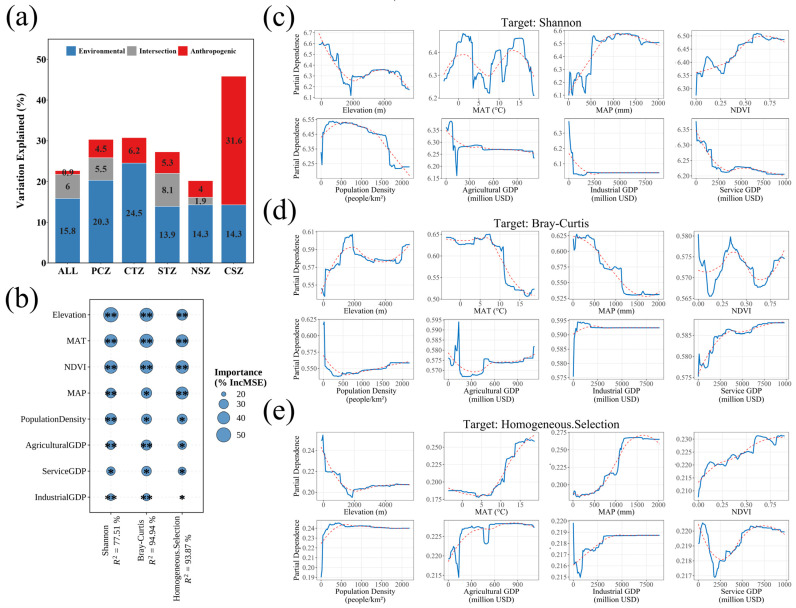
Climatic and anthropogenic drivers of microbial community structure and assembly across climatic zones. (**a**) VPA of environmental and anthropogenic factors; (**b**) importance of factors identified by RF models; (**c**–**e**) PDPs illustrating the relationships between key drivers and microbial indices. The blue solid lines indicate the partial dependence trends, and the red dotted lines indicate the fitted reference trends. Asterisks indicate significance levels: * *p* < 0.05 and ** *p* < 0.01.

To ensure model validity, we first conducted a variance inflation factor (VIF) analysis to assess multicollinearity among the predictor variables. All eight selected environmental and anthropogenic variables yielded VIF values < 5, indicating negligible multicollinearity that does not compromise the interpretation of driver importance (Table S2). Along the continental gradient, the relative influence of climatic versus anthropogenic control on sediment microbiomes shifted significantly. In the PCZ and CTZ, natural environmental factors—including elevation, MAT, MAP, and NDVI—primarily drove community variation, collectively explaining about 20–25% of the total variance. In contrast, the dominance reversed in the subtropical region—particularly the CSZ—where anthropogenic variables explained over 30% of the variance, exceeding the influence of natural drivers (Figure 6a). This shift toward anthropogenic dominance signifies a structural reorganization in the assembly of lake sediment microbiomes, wherein human activities may increasingly rival climatic constraints in shaping community structure.

Random forest analysis identified the most influential environmental and anthropogenic factors, while partial dependence plots elucidated their nonlinear response patterns (Figure 6b–e). Regarding α-diversity (measured by the Shannon index), elevation, NDVI, and MAT emerged as predominant drivers, indicating a joint regulation by energy availability and ecosystem productivity. Species richness declined monotonically with increasing elevation, whereas its relationship with MAT followed a U-shaped pattern, thereby reconciling the observed high diversity in both cold and warm climatic regions [57]. In cold environments, low temperatures may slow competitive exclusion, preserving deep evolutionary lineages—a mechanism consistent with the preservation effect [58]. In contrast, warmer environments likely enhance productivity-driven coexistence, aligning with the species–energy hypothesis [31,32]. NDVI exhibited a positive monotonic relationship with diversity, confirming that vegetative productivity serves as a key determinant of microbial α-diversity [59]. Population density exhibited a hump-shaped association with diversity. This pattern is broadly consistent with evidence that moderate disturbance can increase microbial diversity, whereas stronger urbanization can homogenize microbial communities and reduce alpha diversity [60,61].

Patterns of β-diversity (quantified as Bray–Curtis dissimilarity) revealed a dual structure governed by both physical and anthropogenic barriers. While elevation and MAT remained key constraints, socioeconomic factors—specifically agricultural GDP and population density—exerted comparable influences. This suggests that intensified land use can increase nutrient inputs to lakes and thereby alter sediment-associated microbial dynamics [62]. Community dissimilarity generally increased with elevation, whereas its responses to anthropogenic variables were nonlinear and variable across gradients, suggesting that human activities may reshape community dissimilarity through multiple pathways rather than a single directional trend [10,17]. These non-linear responses highlight a trade-off between dispersal limitation and nutrient-driven homogenization across the climatic continuum [63].

Deterministic community assembly, mediated by homogeneous selection, intensified with energy availability and ecosystem productivity [54]. NDVI, MAT, and MAP emerged as the primary environmental drivers, exhibiting a strong positive correlation with the strength of homogeneous selection. Furthermore, anthropogenic factors—specifically population density and agricultural GDP—also reinforced this trend. The PDPs indicated that increasing anthropogenic disturbance generally enhanced environmental filtering, whereas high anthropogenic intensity was associated with persistently elevated homogeneous selection, potentially favoring communities, predominantly structured by metabolically versatile taxa such as *Bacillus* and Desulfobacterota [46,64]. Collectively, climatic and anthropogenic drivers operated synergistically to shift microbial assembly from stochastic-dominated regimes in cold environments toward deterministic and high-energy regimes characterized by the prevalence of ecological specialization [57].

These findings demonstrated that sediment microbiomes were structured by a non-linear coupling of climatic energy and anthropogenic disturbance. As climatic gradients approach high-energy, high-productivity states, their marginal influence diminished, rendering anthropogenic disturbance decisive in shaping microbial diversity and community assembly. This transition reflected a shift from climatic dominance to coupled regulation, wherein temperature and productivity shaped the broader ecological setting, while land use further fine-tuned community composition [65]. This emergent climate–human synergy indicated a zone of intensified coupled regulation in subtropical systems, marking an increased anthropogenic imprint within the climatic framework of continental aquatic ecosystems.

### 3.5. Microbial Network Architecture and the Trade-Off Between Efficiency and Stability

**Figure 7:**
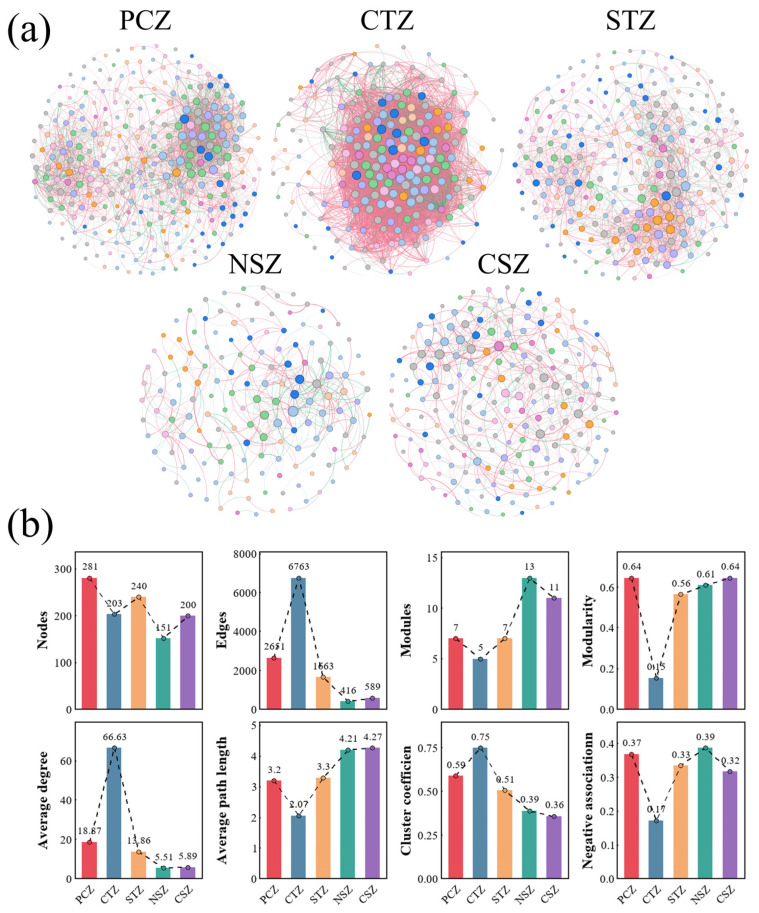
Co-occurrence network structures of sediment microbial communities across climatic zones. (**a**) Genus-level co-occurrence networks in different climatic zones; (**b**) comparison of network topological properties along the climatic gradient, including nodes, edges, modules, modularity, average degree, average path length, clustering coefficient, and negative associations. Different node colors indicate different bacterial phyla.

Climatic gradients exerted pronounced top-down regulatory influence on sediment microbial interaction networks, inducing a systematic structural reorganization—shifting from compartmentalized, persistence-oriented architectures under climatic extremes toward more densely interconnected interaction structures in ecotones. Along the latitudinal gradient, microbial networks exhibited a pronounced U-shaped pattern in modularity and a converse hump-shaped trend in connectivity, suggesting that microbial communities in intermediate temperate zones prioritized highly connected metabolic integration, while climatic extremes favored modular compartmentalization (Figure 7). Zi–Pi role profiling (Table S3) further revealed distinct keystone taxa associated with these regimes. Collectively, these network dynamics reflected the underlying community assembly processes (Section 3.3) and environmental filtering mechanisms (Section 3.4) described above, underscoring a fundamental trade-off between metabolic efficiency and ecosystem stability [66,67].

In the PCZ, network analysis revealed high modularity (modularity = 0.64) and a prevalence of negative associations (37%), consistent with stochastic-dominated assembly processes constrained by cold, oligotrophic conditions. This compartmentalization likely buffered against the propagation of disturbance. Zi–Pi analysis identified stress-tolerant connectors and hubs (e.g., *Candidatus Solibacter*, *Blastocatella*, *Ilumatobacter*), suggesting that plateau stability was underpinned by slow-growing generalists that maintained basal connectivity in nutrient-poor sediments [68]. In contrast, the CTZ exhibited a hyper-connected topology with the highest average degree (66.63) and clustering coefficient (0.75) but minimal modularity (0.15). This intense integration was accompanied by strong dispersal limitation at the community-assembly level (Section 3.3), while the network itself was dominated by positive associations (approximately 83%) [69]. Key connectors, such as *Spirochaeta* and SJA-28, may indicate tighter ecological connectivity, which could favor more integrated community functioning but also increase vulnerability to cascading failures upon disruption of highly connected hubs [68]. The STZ represented an ecological pivot, exhibiting intermediate topology (modularity = 0.56; clustering = 0.51) that indicated a transitional configuration between the highly integrated CTZ network and the more compartmentalized subtropical networks. Zi–Pi results identify *Bdellovibrio* as a key connector, implying that top-down biotic regulation contributed to stability as productivity increased. Furthermore, module hubs affiliated with *Nitrosococcaceae* and *Leptospiraceae* suggested that taxa associated with oxidative and reductive metabolisms co-occurred within a moderately modular framework. In the NSZ, networks were sparsely connected yet highly compartmentalized (modularity = 0.61; average degree = 5.51) and exhibited the highest proportion of negative associations (39%), indicating intensified niche partitioning under warm, productive conditions [70]. Connector taxa (e.g., *Conexibacter*, *Terrimonas*, *Fluviicola*, *Sulfurifustis*) may have linked modules containing taxa associated with carbon- and sulfur-related functions, whereas the presence of only one network hub (Ellin6067) suggested that system-wide cohesion depended on a limited suite of influential taxa. Finally, the CSZ maintained high modularity (modularity = 0.64) but exhibited weak connectivity (average degree = 5.89) and a lower proportion of negative links (32%) compared with the NSZ. Numerous connectors (e.g., *Pelolinea*, *Methylocystis*, Sva0081 sediment group) indicated extensive metabolic bridging, whereas the presence of two hubs (SAR324 and ADurb.Bin063-1) implied a more hub-driven organizational structure under intensified dispersal and anthropogenic homogenization (Section 3.4) [68]. Consequently, CSZ networks exhibited structural resilience through modular redundancy but were more loosely functionally coupled, thereby potentially weakening coordination among taxa associated with different functional guilds [68].

These contrasting network architectures across climatic zones revealed a clear continental-scale pattern: microbial interaction networks shifted from stress-buffering modularity in climatic extremes to densely integrated metabolic coupling in intermediate temperate regions. Synthesizing these continental-scale patterns, we suggest that climatic forcing reorganized microbial interaction networks with a distinct trajectory across the continental gradient. In the cold PCZ, communities exhibited stress-tolerant modularity. As conditions warmed in the temperate CTZ, networks shifted toward high connectivity to optimize metabolic efficiency, while the STZ functioned as a transitional equilibrium. Finally, the subtropical regimes (NSZ–CSZ) became characterized by sparse and modular networks with increasingly segregated interaction structures. The ecological consequences of this topological shift are profound and double-edged. The transition from the hyper-connected networks of the CTZ to the modular, sparse networks of the subtropics suggested a potential trade-off between functional integration and structural resilience [8]. In temperate systems, the tight network connectivity may reflect closer ecological coordination among microbial guilds, potentially facilitating more efficient turnover of shared metabolites [71]. This high connectivity may also be associated with stronger ecosystem processing capacity [72]. However, as climate warming drove these systems toward a subtropical-like modular architecture, this efficient metabolic coordination risked being weakened. The more modular and weakly connected structure observed in the warm, eutrophic CSZ suggested that while the community became more resilient to systemic collapse, ecological interactions may have become less tightly coordinated [16]. The decoupling of strict syntrophic partners allowed functional guilds to operate more independently, which could lead to less coordinated turnover of metabolic intermediates across microbial guilds [19,73]. For instance, the isolation of methanotrophs (e.g., *Methylocystis*) into separate network modules, rather than being tightly integrated with fermenters, may indicate a weaker association between taxa involved in methane-related processes [74]. Consequently, this climatic reorganization may have implications not only for biodiversity but also for the ecosystem’s nutrient buffering capacity and greenhouse-gas-related functions. Thus, the shift from structural connectedness to functional segregation represented a transformation in the ecosystem’s metabolic integrity, making future warmer lakes potentially more resilient in structure but less efficient in function.

### 3.6. A Conceptual Pattern for Interpretation of Sediment Microbiome Organization

**Figure 8:**
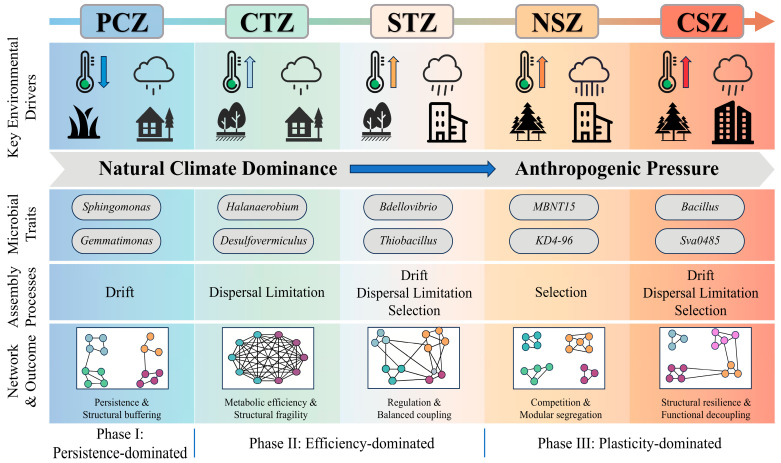
Climate-driven phase transition in sediment microbial community organization along climatic gradients.

By integrating patterns of microbial diversity, community assembly, and network topological structures, we propose a continental-scale conceptual framework: sediment microbiomes were not governed by a single climatic factor but were instead structured by a progressive transition in controlling forces from natural climatic constraints to anthropogenic pressure along an increasing energy–productivity gradient (Figure 8). We further propose a three-stage conceptual pattern of microbial community organization along climatic gradients, characterized by a shift from persistence-dominated to efficiency-dominated, and finally to plasticity-dominated regimes [68,70]. Each regime was defined by coordinated shifts in functional trait syndromes, community assembly rules, and microbial interaction network architectures. This framework summarizes contemporary spatial patterns observed across the climatic zones examined here.

In cold, oligotrophic environments, limited thermal energy and physical isolation imposed strong constraints on microbial growth and dispersal. Under such conditions, ecological stochasticity exerted a dominant influence, selecting for traits associated with stress tolerance and dormancy [75]. The resulting ecological network exhibited pronounced compartmentalization, conferring resilience and structural buffering; specifically, modular organization mitigated the propagation of disturbances and maintained long-term functional continuity under harsh environments, potentially at the expense of less tightly coordinated ecological functioning. Along the transition to temperate conditions, the alleviation of thermal limitation and the development of distinct physicochemical gradients drove community reorganization toward a more efficiency-optimized functional regime. This phase was not governed by a single assembly mechanism; instead, it was shaped by the joint influence of strong spatial constraints and transitional selective effects, which together reconfigured the potential for interaction [27,28]. This transition resulted in more integrated interaction networks with closer potential metabolic coupling, which might have enhanced interaction efficiency while also increasing structural fragility—a canonical trade-off in which tightly coupled systems potentially improved resource transfer and coordination at the cost of heightened sensitivity to perturbations that disrupt key interaction pathways. This efficiency–fragility trade-off in temperate systems set the stage for a further reorganization toward plasticity-dominated regimes as climatic warming became increasingly coupled with anthropogenic forcing.

A critical insight from this study was that microbial organization in the warm subtropics increasingly reflected the coupling between climatic energy and intensifying anthropogenic forcing [76]. Higher productivity and environmentally stressful conditions in the warm subtropics might have favored greater trait plasticity and functional redundancy, with assembly dynamics in the warm subtropics reflecting differentiated mechanisms—stronger homogeneous selection in the NSZ and higher turnover associated with dispersal and drift in the CSZ [77]. Consequently, interaction architecture tended to regain modularity—not as a signature of stress survival, but as a redundancy-supported configuration in which communities were structurally robust yet potentially more functionally decoupled [16]. In this plasticity-driven regime, modularity re-emerged as a form of organizational robustness, but at the cost of weakened cross-guild coordination and reduced efficiency of tightly linked transformations.

This gradient-driven reorganization may have implications for understanding ecosystem responses to global change. Under a space-for-time perspective, warming and accelerating anthropogenic inputs may drive a transition of inland waters toward states characterized by structural resilience but functional decoupling, thereby increasing the risk of functional decoupling and inefficient routing of metabolic intermediates that would otherwise be rapidly processed within tightly coupled consortia [76,78]. Consequently, weakened cross-guild coupling may lead to the transient accumulation of reduced intermediates and labile organic substrates, with possible implications for greenhouse-gas-related processes and episodic water-quality deterioration [76,77]. Simultaneously, the decoupling of remineralization from nutrient transformation may weaken the sediment’s ecological buffering functions and potentially reinforce internal loading feedbacks, rendering eutrophication and hypoxia more persistent under warming regimes [79]. Therefore, future climate-adaptation strategies for inland waters may benefit from moving beyond tracking taxonomic diversity alone. Effective management should prioritize maintaining the interaction integrity of sediment microbiomes—by safeguarding the physicochemical gradients that sustain efficient metabolic coupling where it occurs, and by mitigating nutrient-driven homogenization and persistent hypoxia that promote decoupled, leakage-prone states [80]. Ultimately, our synthesis suggests that the resilience of inland water ecosystems may depend not only on community composition but also on the organization and coupling of microbial interactions under combined climate–human forcing.

## 4. Conclusions

This study provided a continental-scale evaluation of how climatic gradients reorganize sediment microbiomes across lakes in China. Our integrated analysis of microbial diversity, community assembly, and network architecture revealed that sediment microbiomes exhibited nonlinear responses to climatic variation, characterized by a distinct three-stage transition pattern: a persistence-driven assembly regime in the cold plateau regions, an efficiency-driven coupling state in the temperate zones, and a plasticity-driven reorganization phase in the warm subtropical areas.

Crucially, Random Forest models quantitatively identified a critical tipping point in ecological regulation. While microbial communities in plateau and temperate regions were primarily structured by natural climatic and geographic constraints (e.g., temperature, elevation), those in subtropical regions were predominantly governed by anthropogenic forcing. This shift suggests that, within high-energy, productive systems, human activities may have become more influential than natural climate variability in shaping microbial community assembly. Furthermore, the reorganization of interaction networks revealed a fundamental trade-off between biogeochemical efficiency and ecosystem stability. The hyper-connected networks characteristic of the temperate zones may have facilitated tighter ecological integration among microbial guilds, yet still exhibited structural vulnerability to perturbations. In contrast, the modular networks re-emerging in anthropogenically dominated subtropical regions confer structural resilience but might incur functional decoupling—a weakening of syntrophic interactions that could diminish nutrient buffering capacity and enhance greenhouse gas emissions. Consequently, future management of inland waters must move beyond monitoring taxonomic diversity to preserving the organization and coupling of sediment microbiomes. Mitigating nutrient-driven homogenization and hypoxia is essential to prevent these ecosystems from crossing the threshold into decoupled, leakage-prone states, thereby maintaining their vital services in carbon sequestration and water quality regulation under a warming climate.

## Data Availability

The data presented in this study are publicly available in the National Center for Biotechnology Information database, and the corresponding run accession numbers are provided in Table S1 of the Supplementary Materials.

## Supplementary Materials

The following supporting information can be downloaded at: https://www.mdpi.com/article/10.3390/microorganisms14051013/s1. Table S1: The basic information of 562 data points in this study; Table S2: VIF of environmental and anthropogenic variables used in the statistical models; Table S3: Taxonomic classification and topological roles of potential keystone taxa identified by Zi–Pi analysis in sediment microbial co-occurrence networks.
